# Supplementary material for: The evolution of democratic peace in animal societies
Source: Nat Commun. 2024 Aug 3;15:6583. doi: 10.1038/s41467-024-50621-5 (PMC11297998; doi:10.1038/s41467-024-50621-5)
Supplement: Supplementary file 1 — Supplementary Information [file 41467_2024_50621_MOESM1_ESM.pdf]

Supplementary Information

Supplementary Methods 1

To further support the analytical solution described in the main paper, we also provide results from a simulation based on the same model. We initialise a population in which both leaders and followers start with a majority Dove strategy ($P_L = P_F = 0.01$). This initial starting value does not change the model outcome. Using these values, and chosen values for Ω, ε and N (although see below and Supplementary Figure 1 for an explanation of how N makes no qualitative difference to results) we first calculate P, C_L, C_F, V_L and V_F using Equation 1 and the Sharing Rule Equations (Table 2) from the main text. We then proceed to allow leader and follower strategies to update in an iterative process until both classes stabilise on an evolutionarily stable strategy. In each timestep we calculate the relative fitness of playing either Hawk (W_{LH} for leaders and W_{FH} for followers) or Dove (W_{LD} and W_{FD} respectively).

$$\text{Fitness of leader playing hawk } = W_{LH} = P\frac{V_L - C_L}{2} + (1-P)V_L$$

$$\text{Fitness of leader playing dove } = W_{LD} = (1-P)\,\frac{V_L}{2}$$

$$\text{Fitness of follower playing hawk } = W_{FH} = P\frac{V_F - C_F}{2} + (1-P)V_F$$

$$\text{Fitness of follower playing dove } = W_{FD} = (1-P)\,\frac{V_F}{2}$$

If Hawk provides a larger fitness payoff than Dove then the probability of playing Hawk will increase for that class, and vice versa if Dove performs better, the probability of playing Hawk decreases for that class. This iterative process continues until an evolutionarily stable strategy has been found that returns equal fitness between individuals playing Hawk or Dove. Each class's strategy influences the others' (through changes to P; Equation 1 in main text), but both update their hawk-playing probabilities (P_L *and* P_F) independently in each timestep, allowing the model to find a different stable strategy for each class. The amount that the probability of Hawk-playing increases or decreases by in each round is not constant, it decays exponentially in each round, r, governed by the function $0.7e^{(-0.08 * r)}$, such that in the first round the changes are large (0.65) but by the 300th round the increase for successful Hawks or decrease for successful Doves is negligible ($2 * 10^{-11}$). It may seem that we have varied the strength of selection, however this is purely an endeavour of algorithm optimisation. We have added or subtracted incremental and equal changes to strategies of 0.001 in each round (same strength of selection in each round) and find the same results (Supplementary Figure 6). The exact values of 0.7 and 0.08 can be chosen arbitrarily; though these present values have the result that classes converge in a small number of rounds. Strategies have converged even by the 100th round (Supplementary Figure 5), though we run the model to the 300th round to ensure precise results. We find that irrespective of the analytical or simulative methods used, both achieve the same results as described in the main paper.

P_L and P_F are the only two evolvable variables – P is an emergent property which follows from the values of P_L and P_F and other, fixed, parameters. P_L and P_F only depend on P, and $\tilde{V}_L$ and $\tilde{V}_F$. We now show that the value N cancels out from $\tilde{V}_L$and $\tilde{V}_F$, meaning it has no bearing on evolved levels of P_L and P_F. Firstly recall that:

$$V_L = \frac{Vd_v}{N\varepsilon d_v + N(1 - d_v)(1 - \varepsilon)} = \frac{a}{b}$$

$$C_L = \frac{C(1 - d_c)}{Nd_c(1 - \varepsilon) + N\varepsilon(1 - d_c)} = \frac{c}{d}$$

So $\tilde{V}_L = \frac{\frac{a}{b}}{\frac{c}{d}}$ and applying fraction rule $= \frac{ad}{bc}$

$$\tilde{V}_L = \frac{(V \cdot d_v) \cdot (N \cdot d_c \cdot (1-\varepsilon) + N \cdot \varepsilon \cdot (1-d_c))}{(C \cdot (1-d_c)) \cdot (N \cdot \varepsilon \cdot d_v + N \cdot (1-d_v) \cdot (1-\varepsilon))}$$

Factoring out N on both numerator and denominator

$$= \frac{(V \cdot d_v) \cdot N(d_c \cdot (1-\varepsilon) + \varepsilon \cdot (1-d_c))}{(C \cdot (1-d_c)) \cdot N(\varepsilon \cdot d_v + (1-d_v) \cdot (1-\varepsilon))}$$

Cancel the common factor: N

$$= \frac{(V \cdot d_v) \cdot (d_c \cdot (1-\varepsilon) + \varepsilon \cdot (1-d_c))}{(C \cdot (1-d_c)) \cdot (\varepsilon \cdot d_v + (1-d_v) \cdot (1-\varepsilon))}$$

Similarly, for $\tilde{V}_F = \frac{V_F}{C_F}$, recall:

$$V_F = \frac{V(1 - d_v)}{N\varepsilon d_v + N(1 - d_v)(1 - \varepsilon)} = \frac{a}{b}$$

$$C_F = \frac{Cd_c}{Nd_c(1 - \varepsilon) + N\varepsilon(1 - d_c)} = \frac{c}{d}$$

$$\tilde{V}_F = \frac{(V \cdot (1-d_v)\ (N \cdot d_c \cdot (1-\varepsilon) + N \cdot \varepsilon \cdot (1-d_c))}{(N \cdot \varepsilon \cdot d_v + N \cdot (1-d_v) \cdot (1-\varepsilon))(C \cdot d_c)}$$

Factoring out N on both numerator and denominator

$$\tilde{V}_F = \frac{(V(1-d_v) \cdot N \cdot (d_c \cdot (1-\varepsilon) + \varepsilon \cdot (1-d_c))}{N \cdot (\varepsilon \cdot d_v + (1-d_v))(1-\varepsilon))Cd_c}$$

Cancel the common factor: N

$$\tilde{V}_F = \frac{(V(1-d_v) \cdot (d_c \cdot (1-\varepsilon) + \varepsilon \cdot (1-d_c))}{(\varepsilon \cdot d_v + (1-d_v))(1-\varepsilon))Cd_c}$$

The model is thus sensitive to relative proportions of the two classes but not absolute size of the group (see also Supplementary Figure 1).

Supplementary Figures

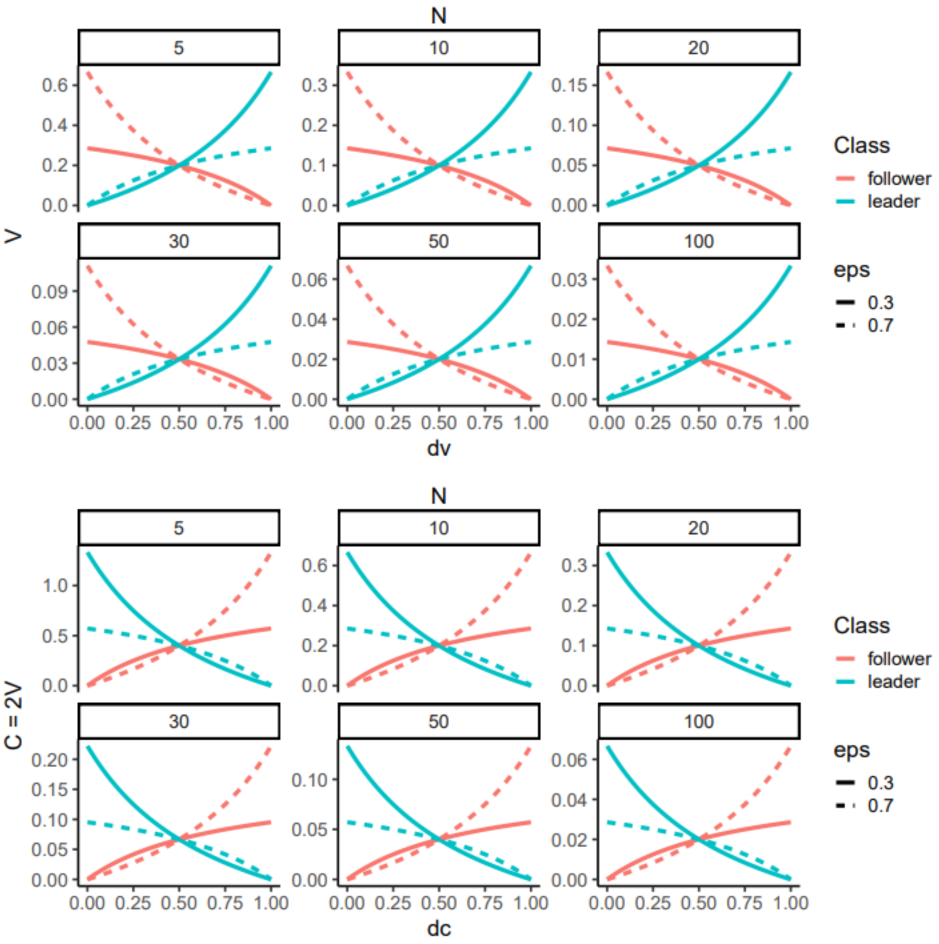

Supplementary Figure 1: The share of benefits *(*top two rows) and costs (bottom two rows) obtained by each individual leader (blue) and follower (pink) under different values of d_v and d_c and *N.* Shares of costs and benefits are calculated using Equations described in Table 2 in Main Text. When $d_v = 0.5$ and $d_c = 0.5$, leader's and follower's receive identical shares, whereas values above 0.5 describe division rules with *advantaged* leaders who pay less of the costs or gain more of the benefits from fighting than followers, and vice versa followers are *advantaged* for values below 0.5. Note that the impact of changing group size (*N*) from 5 (top left within each panel) to 100 (bottom right within each panel) changes the absolute values of each share by rescaling the y-axis but importantly does not change the proportional relationship describing what each leader and follower receives relative to one another. Thus, changing group size (*N*) does not change any of the presented results. Parameter values: $C = 2V$, $d_c = 0.5$ (top row) and $d_v = 0.5$ (bottom row).

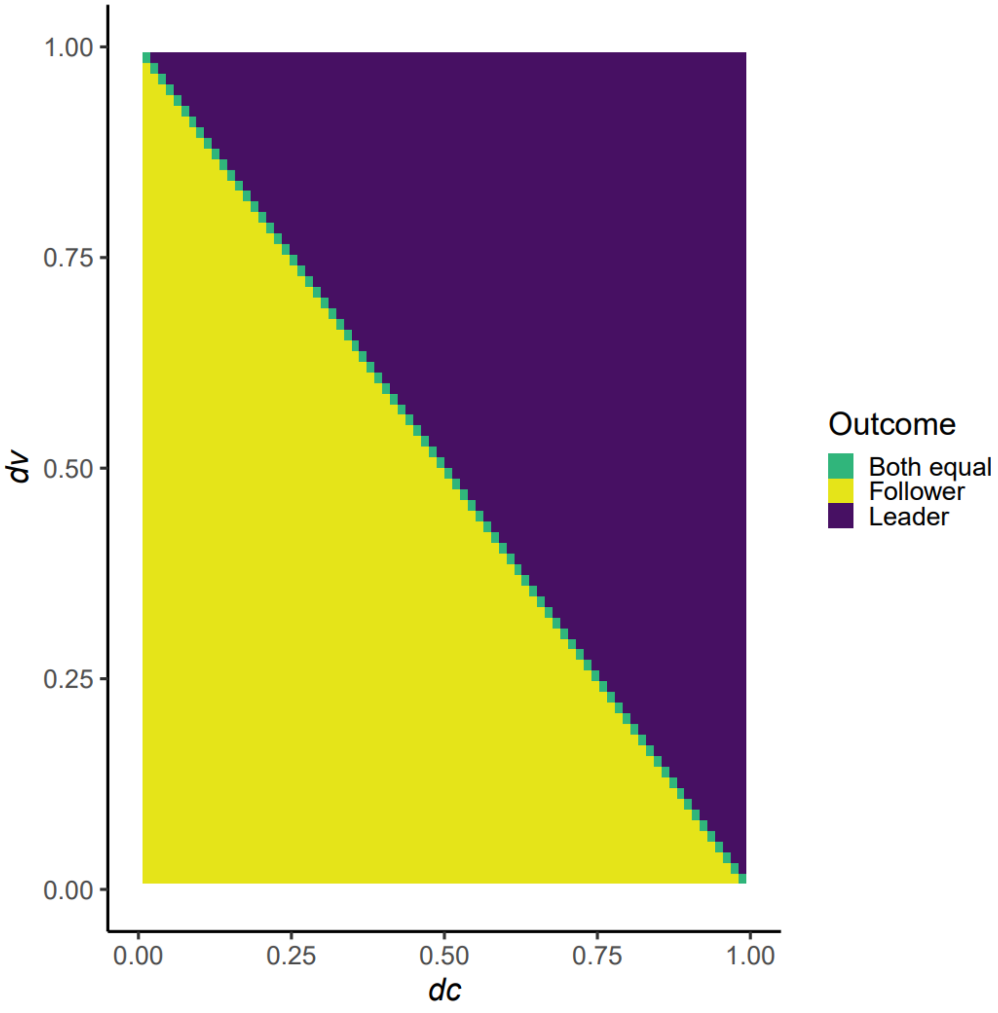

Supplementary Figure 2: Advantaged class have higher optimal Hawk-playing probabilities. Leaders prefer more aggressive interactions by playing Hawk more often than followers ($\tilde{V}_L > \tilde{V}_F$) when $\frac{d_c+d_v}{2} > 0.5$ (shown in purple). Followers have a higher preference than leaders ($\tilde{V}_F > \tilde{V}_L$) when $\frac{d_c+d_v}{2} < 0.5$ (shown in yellow). Leaders and followers have an equal preference ($\tilde{V}_L = \tilde{V}_F$) when $\frac{d_c+d_v}{2} = 0.5$ (shown in green). We use the terms *advantaged* and *disadvantaged* to describe the class which gains more from interactions with outgroups. For example, leaders are described as *advantaged* or *disadvantaged* when the combined sharing rules of d_c and d_v are to the right or left of the diagonal green line respectively.

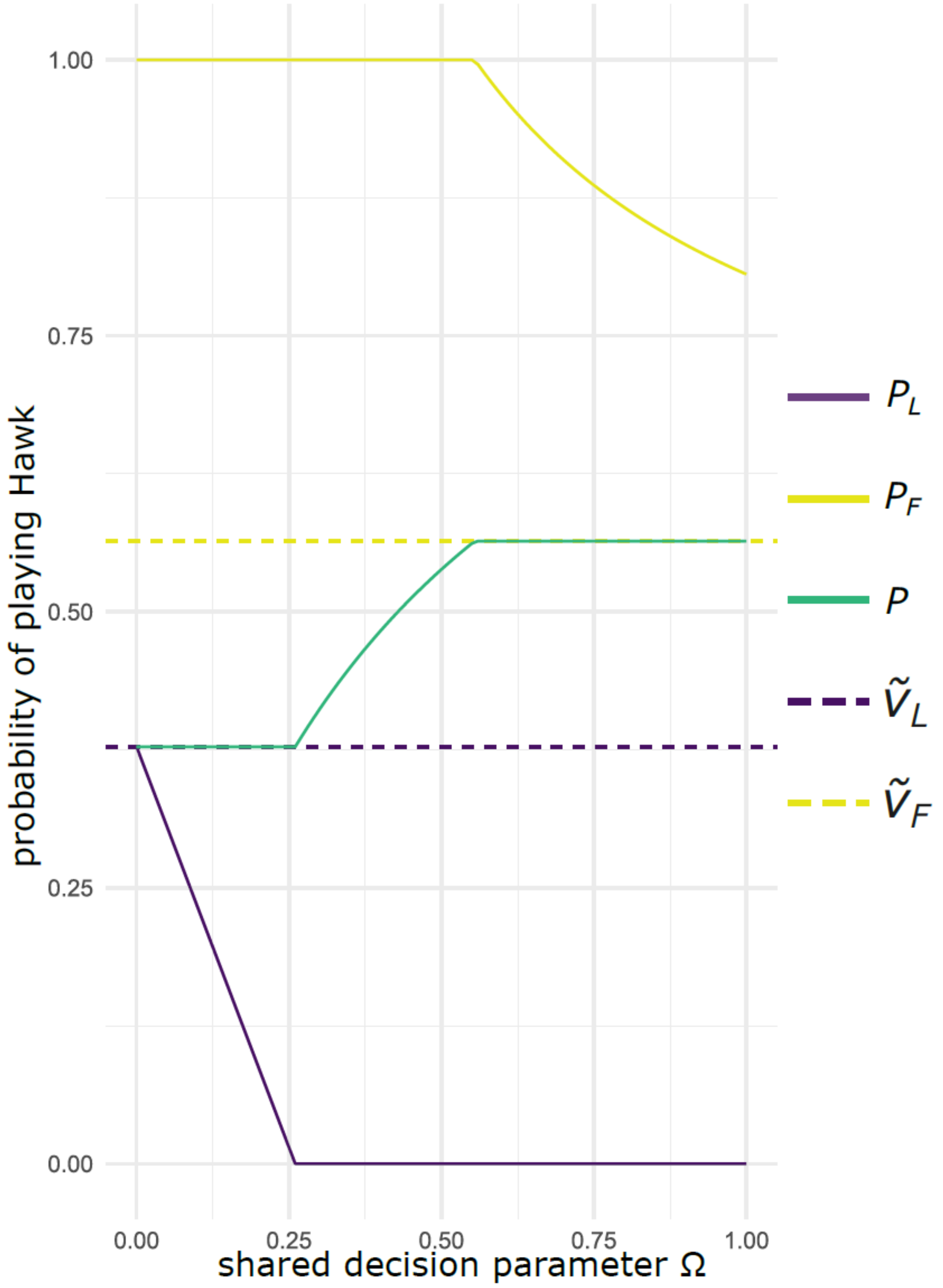

Supplementary Figure 3: Illustrative example of the potential for "democratic war". The propensity to play Hawk for leaders (P_L) and followers (P_F) and the probability of playing Hawk across the population (P) are shown across shared decision parameter (Ω). The population strategy rapidly transitions from leader control ($\tilde{V}_L$) to follower control ($\tilde{V}_F$). Note that hawk playing is higher at the stable strategy under follower control compared to at leader control because followers are advantaged in this example, i.e., $\frac{d_c+d_v}{2} < 0.5$ .Parameter values: ε = 0.3, $C = 2V$, $d_c = 0.45$, $d_v = 0.45$, N = 100.

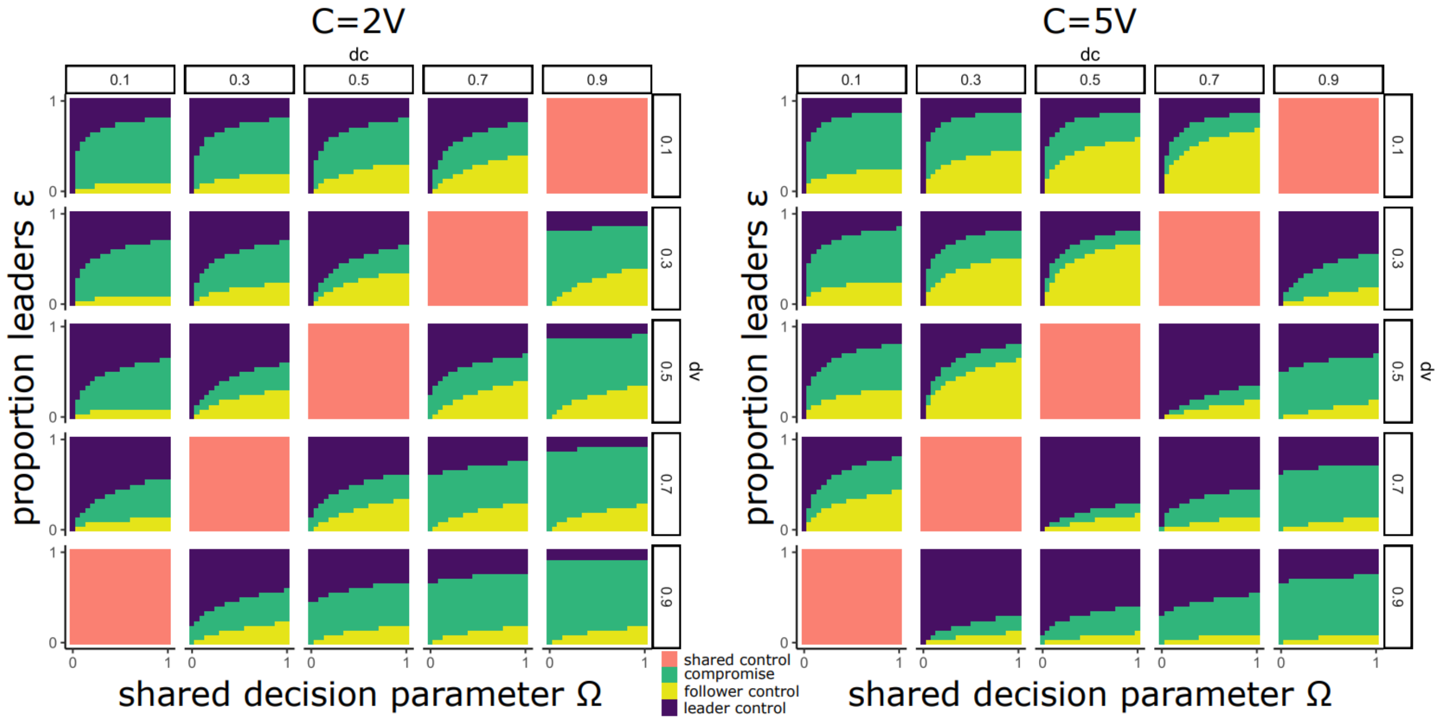

Supplementary Figure 4. Group's evolved strategy in response to Ω and ε also depends on d_v, d_c and C:V ratio. Related to Figure 2 in main paper. Population's evolved strategy as either the stable strategy under follower control ($P = \tilde{V}_F$, shown in yellow), stable strategy under leader control ($P = \tilde{V}_L$, shown in purple), in compromise (P is between $\tilde{V}_F$ and $\tilde{V}_L$, shown in green) or stable strategy under shared control ($P = \tilde{V}_L = \tilde{V}_F$, shown in salmon, please note here that P_L and P_F can differ as long as $P = \tilde{V}_L = \tilde{V}_F$). The share of costs, *dc*, and benefits, *dv* – in which increasing values favour leaders – vary between plots on the x and y axis respectively. Regardless of d_c and d_v, when leaders monopolise decision-making (Ω is low) and when leaders are relatively abundant (ε is high) the group's evolved strategy is likely to be under leader control. When the mean of d_v and d_c are closer to 0 or 1, we see more compromise states (green). This is because there is a greater difference between each classes' stable strategy of Hawk playing, with one classes' strategy close to obligate Dove and the others' close to obligate Hawk. This minimises the anchoring ability of both parties to sway the collective decision in their favour, leading to more compromise outcomes. For more on anchoring see discussion in the main paper. When the mean of d_v and d_c = 0.5, the stable strategy obtained when each class has individual control are both equal ($\tilde{V}_L = \tilde{V}_F$), meaning that they have shared control, but note that each class can still pursue different individual strategies to reach this equilibria, i.e. P_L and P_F can differ as long as $P = \tilde{V}_L = \tilde{V}_F$. Cost of fighting $C = 2V$ in **A** and $C = 5V$ in **B.** The increased cost of fighting decreases the stable strategy of Hawk playing for both parties. It is thus the class which is disadvantaged which lose their anchoring ability. The result is clearly illustrated by the data to the left and right of the salmon outcomes in **B**. To the left, when the followers are advantaged, they have more ability to sway the decision, but leaders have more sway to the right when they are advantaged. This trend is absent in **A** where costs are lower. The higher the costs, the more a class with inherent advantage can turn their higher aggressiveness into influence, though overall strategies are more peaceful. Figure resolution provided at 20x20 data points for each combination of d_v and d_c values.

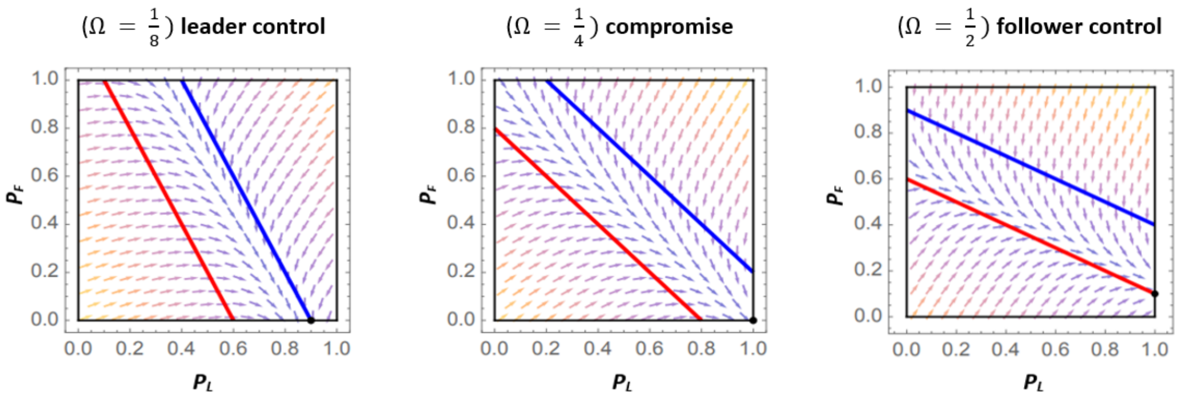

Supplementary Figure 5. Selection on P_L and P_F. Each plot shows the space of possible strategies, with P_L varying along the horizontal axis and P_F along the vertical axis (both ranging from 0 to 1). Arrows show the direction of evolution under the simple adaptive dynamic described in the text. The blue line is the P_L nullcline (i.e. at any point on this line, there is no change in P_L), the red line is the P_F nullcline (i.e. at any point on this line, there is no change in P_F), and the black dot in each case represents the unique equilibrium solution that evolves for the given parameter values. In all three plots, $\tilde{V}_L = 0.6$ and $\tilde{V}_F = 0.4$, implying that leaders are advantaged, and ε = 0.2.

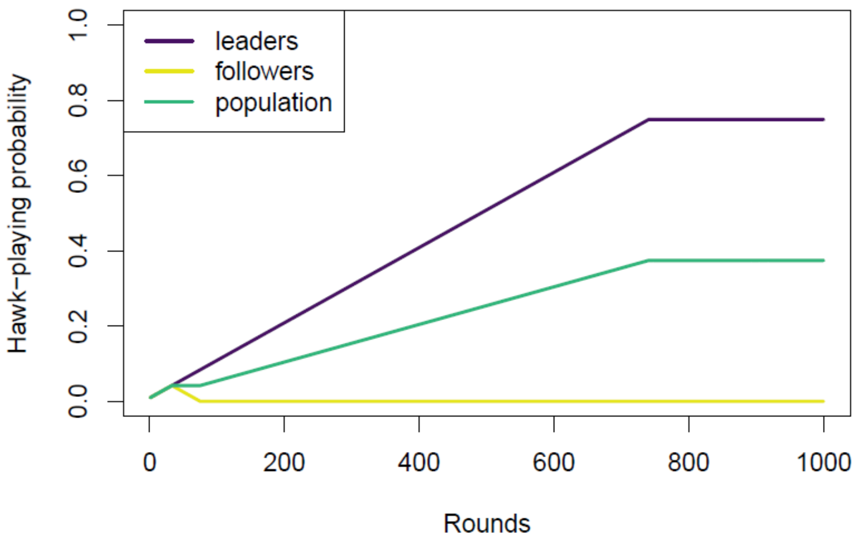

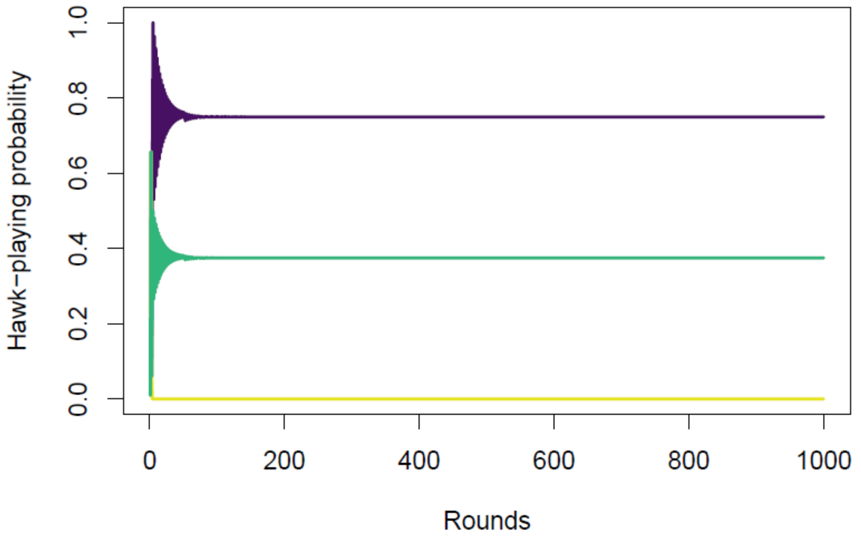

Supplementary Figure 6. Algorithm optimisation for simulative approach. **A)** Method used to generate main results involves a successive decrease in the magnitude used to update the strategy in each progressive round. This allows us to reach the evolutionarily stable strategy faster (N=300 rounds used in main results) than the method in **B)** where strategy differences are always 0.001 in each round. The advantage of our method are savings in computational time and costs, despite obtaining the same end result. Parameter values: $C = 8V$, $d_v = 0.75$, $d_c = 0.75$, $\Omega = 1$, $\varepsilon = 0.5$.
